# Porcine Wound Models: The Gold Standard for Translational Research in Cutaneous Healing

**DOI:** 10.1111/wrr.70204

**Published:** 2026-08-25

**Authors:** Stephen C. Davis, Joshua Tam, Ivan Jozic

**Affiliations:** ^1^ Dr. Phillip Frost Department of Dermatology and Cutaneous Surgery University of Miami Miller School of Medicine Miami Florida USA; ^2^ Wellman Center for Photomedicine, Massachusetts General Hospital & Department of Dermatology Harvard Medical School Boston Massachusetts USA

**Keywords:** biofilm infection, chronic wound modelling, diabetic minipig, full‐thickness wounds, incisional wounds, partial‐thickness wounds, porcine wound models, regenerative medicine, spatial omics, translational research

## Abstract

Porcine wound models are central to translational cutaneous repair research because porcine skin reproduces many structural and healing features of human skin and supports clinically relevant wound sizes, sampling strategies and device testing. This review summarises the historical development and current use of major porcine wound platforms including incisional, partial‐thickness, full‐thickness, infected, chronic‐like, diabetic, ischaemic, burn, pressure ulcer, hypertrophic scar and radiation models, with emphasis on methodological rigour, translational strengths and study‐specific limitations. We further highlight how spatially resolved molecular profiling, longitudinal imaging and integrated transcriptomic datasets are expanding mechanistic interrogation of porcine wounds and may support development of AI‐enabled analytic frameworks when linked to well‐annotated healing outcomes. Emerging humanised and genetically engineered swine provide additional opportunities to model human‐specific immune or comorbidity contexts, although broader validation remains necessary. Key challenges remain the difficulty of sustaining chronicity in otherwise healthy animals, inter‐study variability in wound generation and reporting and the need to align preclinical endpoints with clinically meaningful outcomes. Overall, porcine models remain the most clinically relevant large‐animal platforms for bridging mechanistic wound‐healing studies and late‐stage preclinical therapeutic evaluation.

## 1 Introduction

**FIGURE 1:**
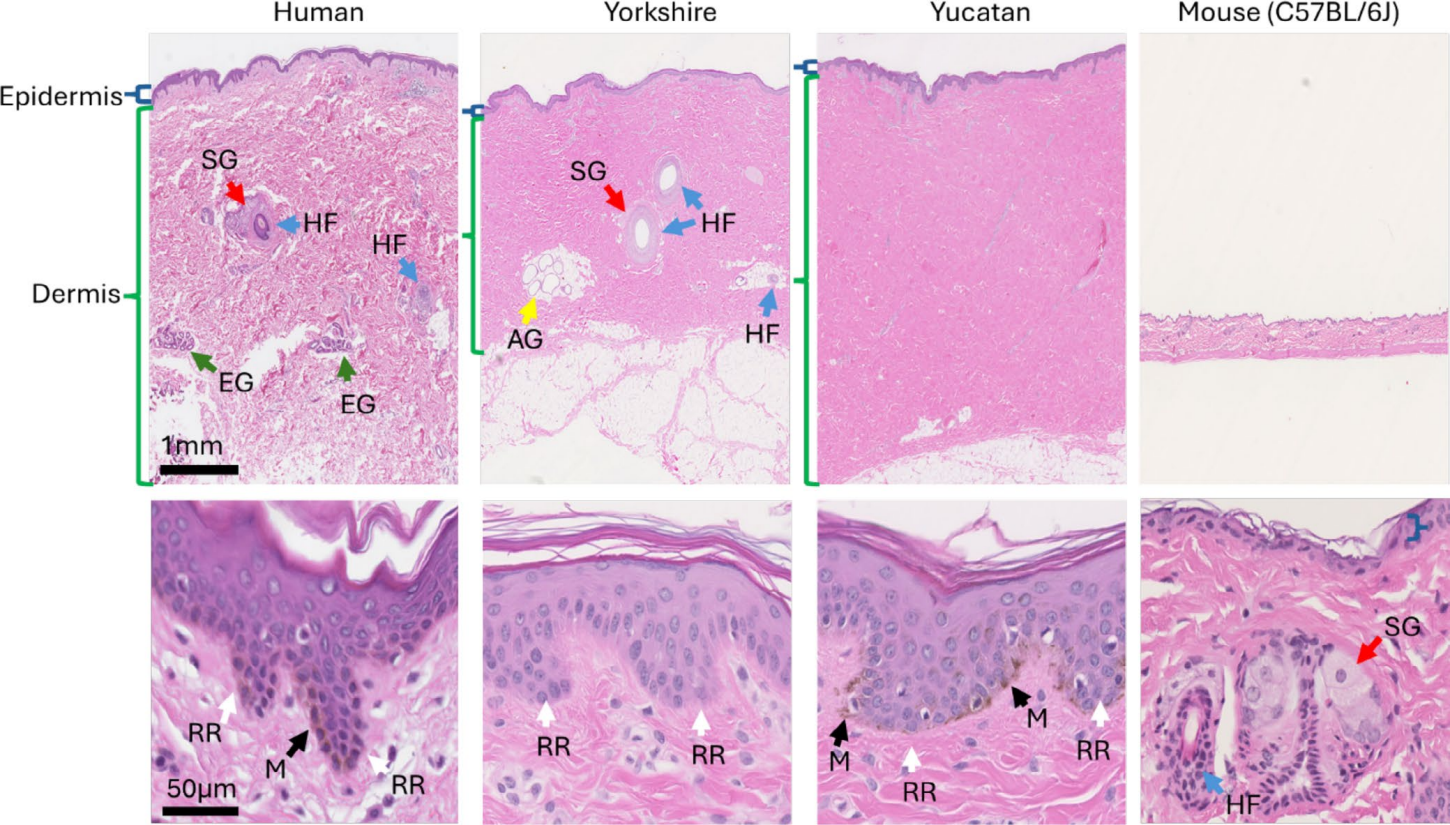
Comparative histology of human, porcine and murine skin. Representative histological sections of human, porcine (Yorkshire and Yucatan) and murine (C57BL/6J) skin illustrating key similarities and species‐specific differences in skin architecture. Upper panels show low‐magnification views highlighting overall epidermal and dermal thickness and organisation, while lower panels show higher‐magnification views of epidermal‐dermal junctional features. Human and porcine skin display a relatively thick epidermis and dermis with well‐defined rete ridges (RR) and adnexal structures, whereas murine skin is thinner and densely populated by hair follicles (HF). Sebaceous glands (SG), eccrine glands (EG) and apocrine glands (AG) are evident in human and porcine skin, with porcine skin showing closer architectural resemblance to human skin than murine skin. Melanocytes (M) and RR morphology differ across species, reflecting distinct epidermal organisation and barrier characteristics. Scale bars: 1 mm (upper panels); 50 μm (lower panels).

The study of wound healing has long depended on animal models to bridge mechanistic insight and clinical application. Among available preclinical systems, the porcine model has emerged as a highly translational platform for cutaneous wound‐healing research because porcine skin shares important anatomical, physiological and immunological features with human skin. Comparative histological and immunohistochemical analyses have demonstrated that porcine skin closely mirrors human skin in epidermal thickness (30‐140 μm in pigs vs. 50‐120 μm in humans), dermal architecture, collagen organisation, vascularisation and immune cell composition [1, 2, 3, 4]. The dermal‐epidermal thickness ratio (~10:1‐13:1), presence of rete ridges and distribution of dendritic and Langerhans cells further reinforce this similarity [1, 3, 5, 6]. These features enable pigs to model wound‐healing responses and tissue repair patterns more faithfully than small‐animal models. Rodent systems, while valuable because of cost and genetic tractability, exhibit important limitations. Rodents heal primarily through contraction, driven by the panniculus carnosus muscle, a mechanism largely absent in humans [7, 8, 9]. Their skin is thinner, more loosely attached and structurally distinct, with a higher density of hair follicles and immune features that incompletely reflect human inflammatory pathways [8, 10]. These differences limit translational fidelity, particularly for studies of chronic wounds, fibrosis and immune‐mediated healing [9, 10, 11] (Figure 1).

Over the years, porcine platforms have become instrumental in evaluating advanced dressings, antimicrobial strategies, biomaterial scaffolds and device‐based therapies. Foundational studies using pigs led to the development of moist wound‐healing protocols [12], while preclinical porcine data informed the clinical adoption of negative‐pressure wound therapy (NPWT) [13]. A landmark comparative review also reported higher concordance between preclinical porcine findings and human clinical outcomes than was observed in rodent models, supporting the predictive value of porcine systems for late‐stage translational testing [14].

This review synthesises the evolution, methodology and translational utility of major porcine wound models, from incisional and excisional paradigms to infected, chronic‐like and diabetic systems. We further highlight emerging and specialised models for hypertrophic scarring, thermal injury, radiation dermatitis and pressure ulcers and discuss how bulk and spatially resolved molecular profiling, including transcriptomic and proteomic approaches, together with longitudinal imaging and computational analysis, may strengthen mechanistic insight and improve identification of pathways relevant to impaired healing and therapeutic targeting. Finally, we outline best practices for standardisation and propose future directions (including AI‐enabled analytic approaches and humanised or genetically engineered porcine systems) to strengthen the pipeline from preclinical validation to clinical translation.

### 1.1 Historical Perspective: The Evolution of Porcine Wound Models

Porcine wound models were adopted in the mid‐20th century as investigators sought preclinical systems with human‐like skin structure and clinically relevant wound dimensions. A landmark 1962 study in young pigs demonstrated that moist wound environments promote faster re‐epithelialisation than dry conditions, helping establish the biologic rationale for occlusive dressings [12]. Building on this foundation, subsequent quantitative porcine wound assays enabled more rigorous testing of therapeutic strategies. In 1978, superficial wounds covered with a polyethylene film showed accelerated epidermal healing, whereas topical corticosteroid treatment delayed epithelial closure, illustrating how both occlusion and anti‐inflammatory agents can modulate repair [15]. The following year, the same general platform was applied to topical antimicrobials, showing that some agents modestly improved closure whereas others were neutral or detrimental [16]. Together, these early studies demonstrated the sensitivity of porcine wounds to treatment‐related differences and helped establish the model as a practical platform for preclinical wound‐care evaluation.

By the early 1980s, Eaglstein and colleagues had not only confirmed the benefits of occlusion [17, 18, 19] but had also shown that even seemingly inert treatment vehicles could influence wound closure. In a 1980 study, white petrolatum delayed epidermal healing in superficial pig wounds, whereas an oil‐in‐water vanishing cream accelerated healing relative to untreated controls, emphasising that vehicles are not biologically inert [20]. Such findings underscored the importance of appropriate control groups and informed formulation of topical therapeutics thereafter. Around the same time, efforts to model wound contamination in vivo led to a standardised porcine partial‐thickness infection assay showing that certain antiseptics could reduce bacterial burden without impairing healing [21, 22]. These studies helped define the porcine model not only as a platform for evaluating dressings and topical formulations, but also as an early system for studying contamination control and antimicrobial intervention.

In the 1990s, the porcine model became a platform for both clinically relevant intervention testing and mechanistic discovery. A notable advance in burn wound management came from a pig model showing that early surgical debridement improved healing outcomes, supporting the clinical rationale for early burn excision [23]. Around the same time, swine models were used to establish the experimental basis for NPWT, demonstrating increased local blood flow, enhanced granulation tissue formation and reduced bacterial burden under controlled suction conditions [13]. By the late 1990s, porcine systems were also being applied to dermatologic procedures and fibrosis‐related interventions. Comparative studies of post‐operative care after laser resurfacing showed that dressing choice influenced epidermal regeneration and collagen remodelling [24] while full‐thickness wound studies using TGF‐β2 and neutralising antibodies demonstrated predictable changes in collagen alignment relevant to scar biology [25]. Together, these studies illustrate how porcine models evolved into versatile systems for both therapeutic evaluation and biologic insight.

Subsequent comparative analyses and increasing methodological standardisation strengthened the rationale for porcine studies as late‐stage preclinical tests of wound therapeutics. Comparative evaluation across animal models showed that porcine wound‐healing findings correlated more closely with human clinical outcomes than rodent data, reinforcing the clinical relevance of porcine systems [14]. At the same time, porcine models were extended to more challenging pathophysiologic settings. Deep dermal burn models were used to generate hypertrophic scars [26], streptozotocin (STZ)‐induced hyperglycaemia was used to model diabetic impairment [27], and bipedicle flap paradigms were developed to reproduce chronic ischaemic wound environments [28]. These advances expanded porcine wound research beyond acute repair and into the study of hard‐to‐heal phenotypes under clinically relevant biologic constraints.

In parallel with these specialised models, porcine wound research in the 2000s expanded substantially into infection and biofilm biology. A major advance was the direct in vivo visualisation of wound biofilm in porcine wounds inoculated with *Pseudomonas aeruginosa* , demonstrating bacterial aggregates embedded in extracellular matrix and linked to delayed healing [29]. Subsequent polymicrobial infection studies further showed that combined MRSA and *P. aeruginosa* infection produced more severe biofilm formation, stronger inflammatory responses and slower closure than single‐species infection [30]. These findings helped establish porcine infection models as clinically relevant systems for studying bacterial pathogenesis, biofilm persistence and antimicrobial intervention strategies.

By the 2010s, the pivotal role of porcine models in advancing wound‐healing science was widely recognised, and attention increasingly turned toward consolidation, standardisation and higher‐resolution analytic integration. Thought leaders in wound research called for rigorous methodological standards to maximise the translational yield of pig studies, emphasising that while rodent models remain invaluable for genetic and mechanistic discovery, large‐animal models (especially pigs) are often indispensable for bridging to human trials [9, 11, 31]. This period also saw increasing incorporation of high‐resolution imaging, transcriptomics and proteomics into pig studies to dissect the molecular underpinnings of wound healing and to link conventional histologic endpoints with higher‐dimensional molecular readouts, further strengthening the human‐pig translational link and underscoring the need for standardised protocols, data sharing and reporting frameworks such as ARRIVE 2.0 to improve reproducibility and regulatory relevance [10, 32, 33].

**FIGURE 2:**
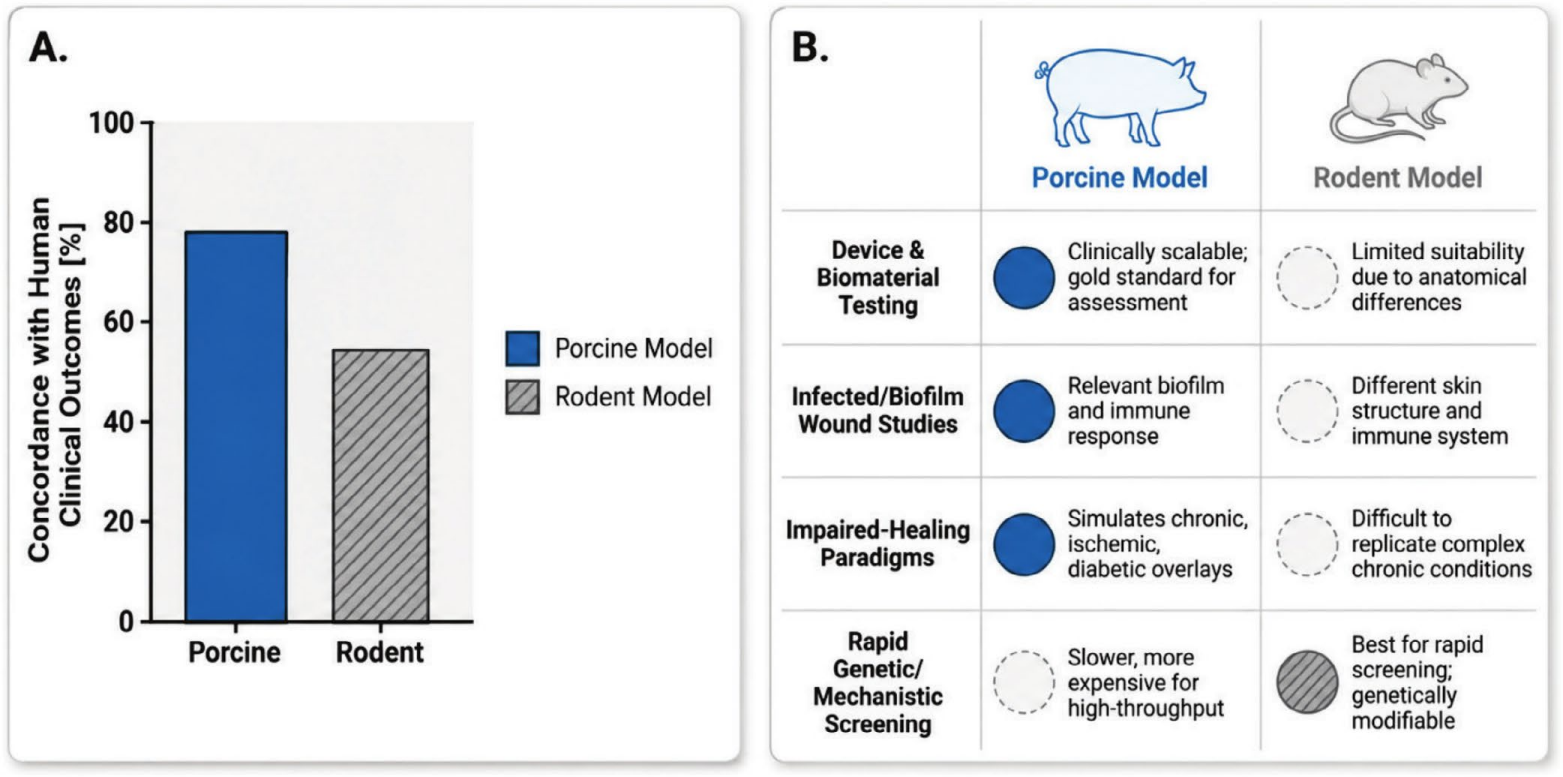
Porcine skin demonstrates superior translatability for evaluating wound healing therapies and biomaterials. (A) Bar graph comparing the concordance of preclinical animal models with human clinical wound‐healing outcomes derived from Sullivan et al. [14] which demonstrate that porcine models achieve approximately 78% predictive agreement with human trials, whereas rodent models (mice/rats) show approximately 50%–60% concordance.

Over the span of five decades, the continual refinement of porcine wound models has transformed them into a broad toolkit for translational research. Porcine systems now encompass acute surgical wounds, superficial abrasions, deep excisions, burns with hypertrophic scarring, infected and biofilm‐associated wounds, ischaemic wounds, diabetic wounds and multifactorial impaired‐healing states. From early experiments that helped establish modern moist‐wound‐healing concepts to contemporary studies integrating molecular profiling and advanced imaging, this progression has reinforced the central translational value of porcine models. The historical development of the field therefore provides not only context for current model selection but also a basis for future innovation in wound modelling and therapy development. A timeline summarising key milestones in the development of porcine wound‐healing models is provided in Figure 2.

## 2 Core Porcine Wound Models and Their Applications

**FIGURE 3:**
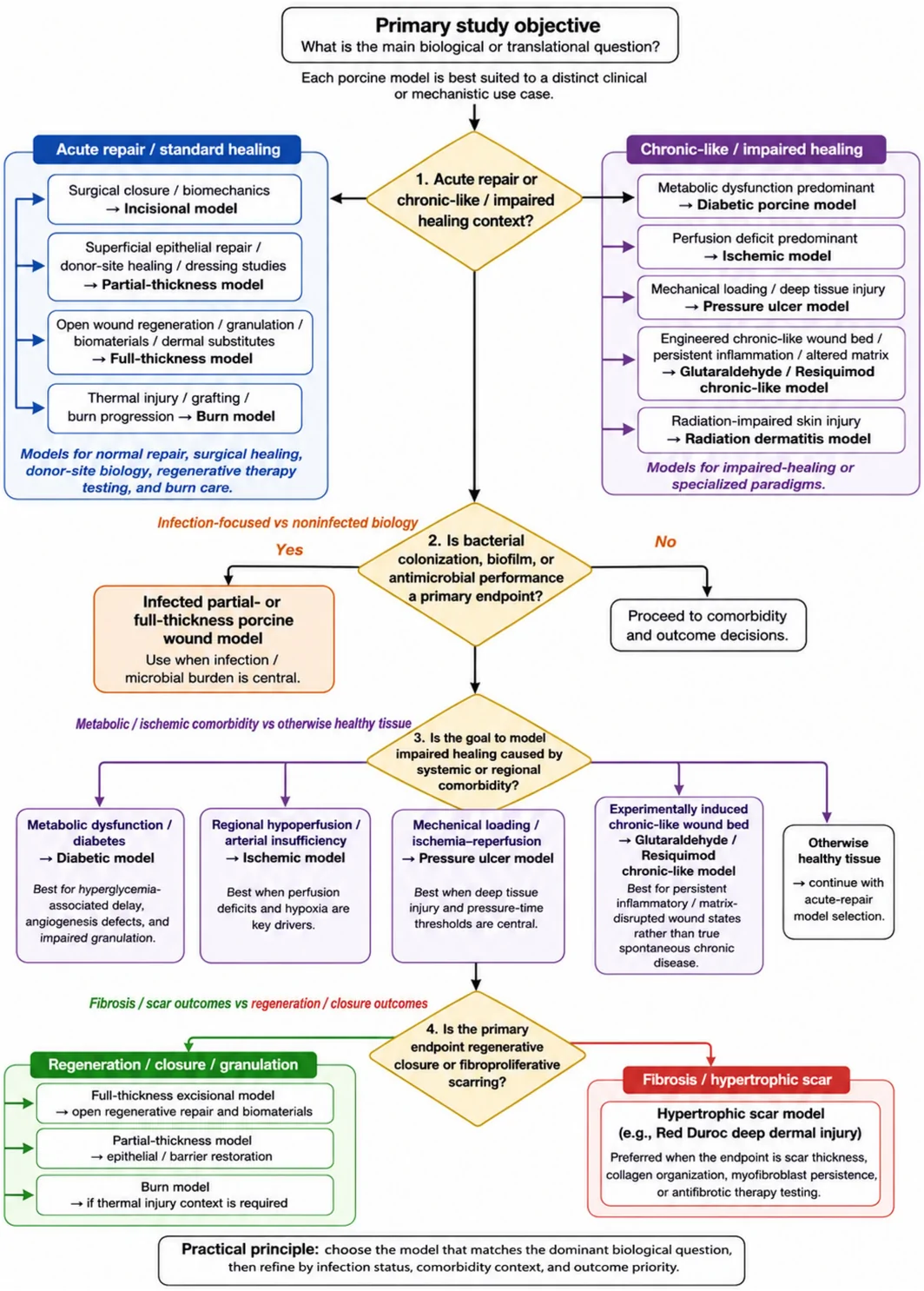
Decision frameworks for selecting porcine wound models based on the primary translational question.

Below, we summarise the major porcine wound models, each aligned with a common clinical scenario, together with their methodology, translational rationale, representative applications, principal strengths and key limitations. Where relevant, recent innovations such as advanced imaging, molecular profiling and combined comorbidity overlays are also noted (Table 1, Figure 3).

**TABLE 1:** Core porcine wound models.

Model	Typical methodology/key features	Common primary endpoints	Translational utility	Representative references
Incisional	Linear full‐thickness skin incisions closed with sutures or staples; supports paired within‐animal comparisons and biomechanical testing.	Tensile strength; wound integrity; re‐epithelialisation; collagen organisation; scar width.	Surgical wound healing, closure devices, incision dressings and scar‐modifying interventions.	[5, 34, 35, 36, 37]
Partial‐thickness excisional	Dermatome‐generated wounds with preservation of part of the dermis and adnexal structures; requires careful depth calibration and depth confirmation. Site‐ and sex‐related dermal thickness variation should be considered	Time to re‐epithelialisation; epithelial migration; barrier recovery (e.g., TEWL); epidermal thickness/differentiation; superficial perfusion/imaging metrics	Donor‐site and superficial wound healing; dressings, topical agents, epidermal repair and barrier‐restoration studies	[1, 2, 3, 4, 5, 6, 12, 15, 18, 38, 39, 40, 41, 42]
Full‐thickness excisional	Excision through the full dermis to subcutaneous fat or fascia; wound depth and termination plane should be reported explicitly because retained adipose tissue can affect wound‐bed composition	Closure kinetics; granulation tissue thickness/quality; angiogenesis; inflammatory infiltrates; collagen deposition; re‐epithelialisation; scar outcome	Regenerative therapies, biomaterials, dermal substitutes, angiogenic therapies, fibrosis/scarring studies and late preclinical testing	[9, 14, 31, 43, 44, 45, 46, 47, 48, 49, 50, 51, 52, 53, 54, 55, 56, 57, 58]
Infected/biofilm	Partial‐ or full‐thickness wounds inoculated with defined organisms (e.g., *S. aureus* , MRSA, *P. aeruginosa* , polymicrobial consortia) under controlled occlusion. Strict aseptic techniques are required to prevent cross‐wound contamination	Quantitative bacteriology; biofilm imaging; inflammatory histology; delayed closure; epithelial migration; exudate/tissue sampling	Antimicrobial, antibiofilm, debridement and infection‐control studies in a clinically relevant large‐animal setting	[21, 29, 30, 59, 60, 61, 62, 63, 64, 65]
Burn/Thermal	Standardised contact or scald injury with controlled temperature, pressure and dwell time; can model partial‐thickness, full‐thickness, excision/grafting and hypertrophic scar outcomes.	Burn depth progression; epithelial closure; infection; graft take; scar thickness; collagen organisation; inflammatory and transcriptomic responses	Burn progression, excision strategies, skin substitutes, infection control, fibroproliferative scarring and regenerative cell‐based therapies.	[23, 26, 66, 67, 68, 69, 70, 71, 72, 73, 74]
Diabetic	STZ‐induced hyperglycaemia or diet‐/genotype‐related metabolic dysfunction in minipigs, followed by standardised excisional wounds.	Delayed closure; angiogenesis defects; impaired granulation; prolonged inflammation; infection susceptibility; microvascular and molecular readouts	Impaired‐healing studies relevant to diabetic wounds and testing of biologic, biomaterial, metabolic and vascular‐targeted therapies	[27, 31, 70, 71, 72, 73, 75, 76, 77, 78, 79, 80]
Ischemic	Local perfusion impairment, commonly using a bipedicle dorsal skin flap with paired ischemic and non‐ischemic wounds in the same animal.	Perfusion/oxygenation metrics; closure delay; granulation tissue; capillary density; hypoxia‐associated markers; tissue strength.	Arterial insufficiency‐type wound healing, hypoxia biology and testing of pro‐angiogenic or oxygen‐based interventions.	[14, 28, 48, 74, 81, 82, 83]
Pressure ulcer/pressure injury	Controlled pressure loading, often with ischaemia–reperfusion components and regulated microclimate, to induce deep tissue injury and ulceration	Tissue injury thresholds; ulcer formation; deep tissue damage; reperfusion injury; healing delay	Pressure‐injury pathophysiology, prevention strategies, pressure‐redistribution devices and microclimate studies	[4, 31, 78, 84, 85, 86, 87, 88]
Hypertrophic scar	Deep dermal injury, especially in scar‐prone breeds such as Red Duroc pigs, followed by longitudinal scar maturation assessment.	Scar height/thickness; collagen organisation; myofibroblasts; pigmentation/erythema; profibrotic signalling	Fibroproliferative scarring mechanisms and preclinical testing of anti‐scarring therapies	[46, 66, 67, 68, 69, 89, 90, 91, 92, 93, 94, 95, 96, 97]

### 2.1 Incisional Porcine Wound Models

**FIGURE 4:**
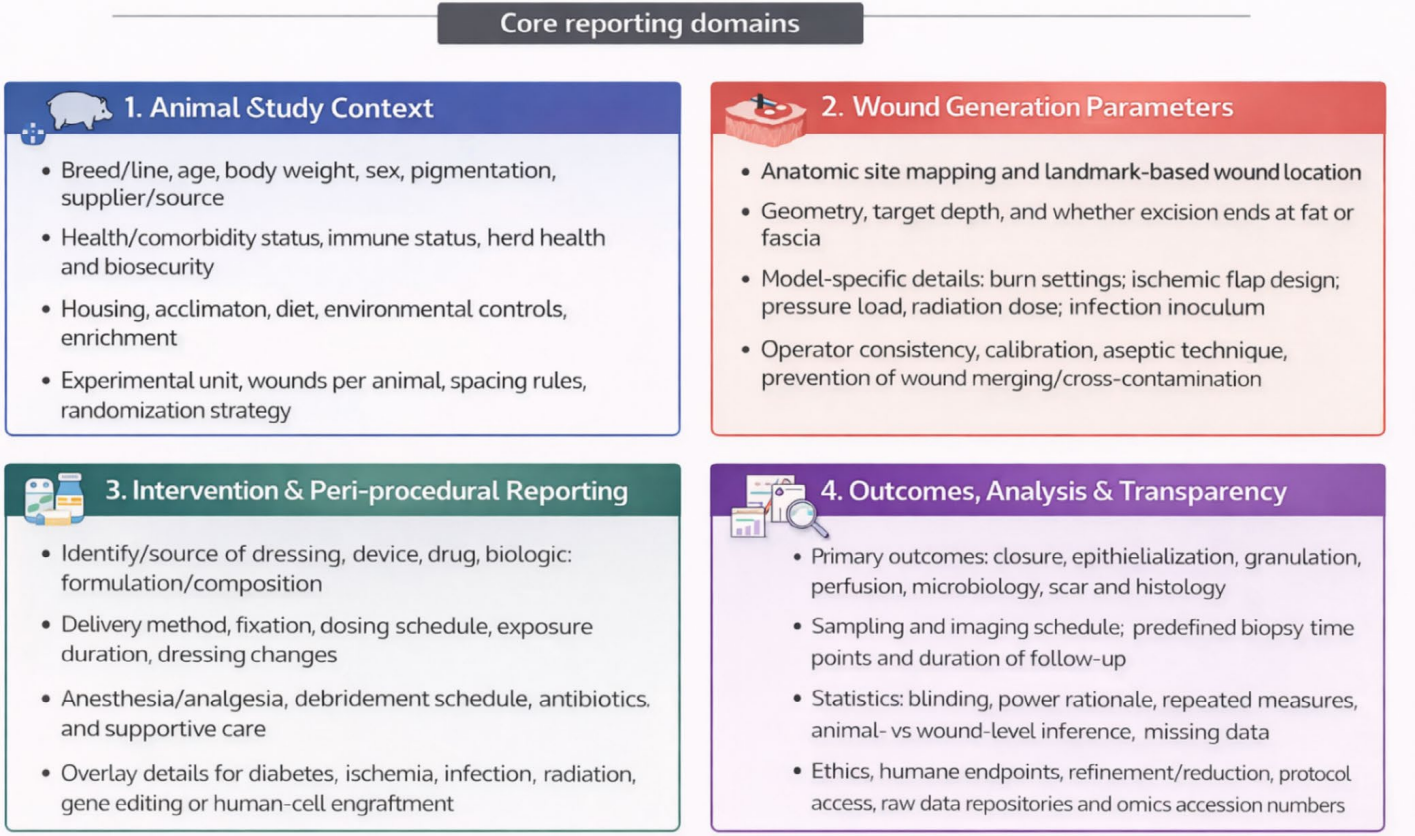
Essential reporting domains and key elements for reproducible porcine wound research.

**FIGURE 5:**
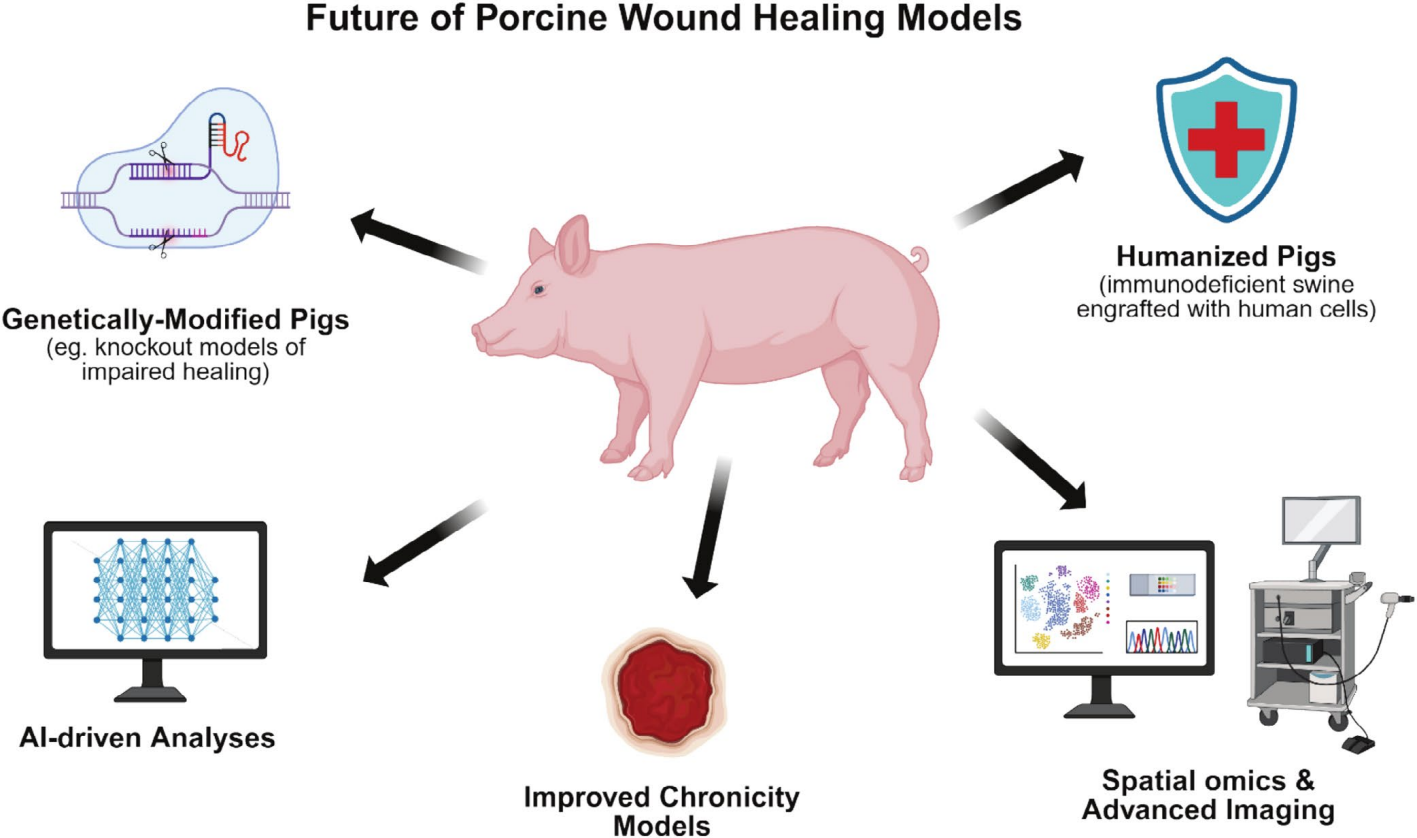
Future directions for porcine wound models. Summary of emerging innovations in porcine wound‐healing research in effort to enhance the translational power and biological relevance of porcine wound models. *Genetically‐modified animals*, enabling gene‐edited or disease‐mimicking models for studying impaired healing; *Humanised pigs*, including immunodeficient swine engrafted with human cells to allow evaluation of human‐specific immune responses; *Spatial omics and advanced imaging*, integrating high‐resolution transcriptomics, proteomics and live microscopy to map healing dynamics; *AI‐driven wound analysis*, applying machine learning to imaging and molecular datasets for predictive modelling; and *Improved chronicity models*, including chemical, ischemic and biofilm‐based methods for producing stable non‐healing wounds.

Incisional wounds are linear skin injuries, typically closed with sutures or staples, that are used to model surgical incision healing, wound biomechanics and surface epithelial repair. In swine, incisional wounds are commonly created on dorsal or flank skin using scalpels or thermal devices such as electrocautery, allowing comparison of healing under different forms of tissue injury [5, 34]. After closure with interrupted or running sutures, key outcomes include tensile strength, wound‐gap integrity, histologic re‐epithelialisation and scar formation [35]. This model permits evaluation of closure materials and incision‐support devices at a clinically relevant scale. Because contraction is relatively limited in pigs, the model is particularly useful for comparing closure techniques and adjunctive interventions that depend on incision integrity [34, 35]. Incisions in pigs also re‐epithelialise rapidly and undergo collagen remodelling in a manner broadly similar to humans [14]. By contrast, rodent models depend much more heavily on wound contraction, which limits their utility for evaluating surgical closure approaches and incision‐support technologies (Figures 4 and 5).

Technically, multiple incisions can be created on a single animal, enabling paired and randomised study designs in which each incision serves as its own control [31]. Incisions are typically made through the full thickness of the skin but usually not into underlying muscle unless dead space is intentionally incorporated. Common variants include sharp scalpel incisions, thermal or electrocautery incisions and laser‐based incisions, which allow comparative evaluation of different injury modalities [34, 36, 37]. Tensile strength is generally measured by mechanical testing of healed skin strips, while histology is used to assess epidermal closure, collagen orientation and the extent of any granulation tissue present.

The incisional porcine model has been widely used to evaluate interventions relevant to surgical wound management, and several consistent themes have emerged across decades of research. This platform is particularly well suited for studying how topical agents, dressings and surgical techniques influence early epithelial closure, collagen organisation and the mechanical strength of healing incisions. Studies using this model have shown that even seemingly inert topical formulations can measurably accelerate or delay epidermal resurfacing, highlighting the sensitivity of incisional wounds to surface chemistry and moisture balance [20]. The model also supports assessment of postoperative incision‐management technologies, including prophylactic NPWT, which has been associated with reduced fluid accumulation beneath closed incisions, improved early perfusion and higher tensile strength during the first week of healing [34, 37]. In addition, the porcine incision model has been useful for mechanistic studies of fibrosis and scar modulation, demonstrating that perturbation of pathways such as TGFβ signalling produces predictable changes in collagen alignment and scar stiffness [25]. Collectively, these observations support the use of the incisional porcine model for evaluating surgical closure materials, adjunctive therapies and molecular regulators of incisional healing and scarring.

The incisional pig model offers several important strengths: it reproduces key biomechanical and histologic features of human surgical wounds, including endpoints such as tensile strength and scar width that relate closely to clinical healing quality. Reproducibility is enhanced by the ability to create multiple incisions per animal and by the use of full‐size surgical materials and devices. However, the model also has important limitations. Healthy pig incisions close rapidly and predictably, which reduces utility for studying chronic or pathophysiologically impaired healing. In addition, the model generates relatively little granulation tissue, making it less informative for therapies directed at deeper wound beds. Outcomes are also sensitive to closure technique and suture tension, underscoring the need for rigorous standardisation. Ethical and cost considerations remain relevant, although incision models are generally less disruptive than open excisional wounds.

### 2.2 Partial‐Thickness Excisional Porcine Wound Models

Partial‐thickness wounds involve controlled removal of the epidermis and part of the dermis, while preserving adnexal structures such as hair follicles and glands that serve as keratinocyte reservoirs for rapid re‐epithelialisation. In pigs, these wounds are typically produced using a calibrated dermatome set to approximately 0.4‐0.5 mm depth, yielding a uniform superficial defect comparable to human donor‐site wounds or superficial partial‐thickness burns [1, 2, 15]. The dorsolateral back is generally used to ensure consistent surface geometry and to allow creation of multiple standardised test sites. Because healing kinetics strongly depend on the amount of residual dermis, precise depth control is essential, often confirmed by imaging or histology [6, 26, 29, 30, 38, 98, 99]. Healing time is strongly dependent on residual dermis; therefore, dermatome calibration and depth confirmation (histology or imaging) are critical for comparability across studies. In addition, empirical depth estimation should account for the wound site and animal characteristics, including sex and regional variation in dermal thickness, because residual dermis can vary across the dorsum and influence healing kinetics. Shallower wounds typically close within ~1 week, whereas deeper partial‐thickness wounds require longer. These findings reinforce the need for depth standardisation in preclinical studies to ensure reliable comparison across interventions [3, 5, 14].

Healing proceeds through migration and proliferation of keratinocytes from adnexal structures within the wound bed and wound edges, producing an epithelial‐based mode of repair that closely mirrors human partial‐thickness wound healing. This makes the model particularly well suited for evaluating dressings, topical agents and regenerative strategies intended to accelerate epidermal coverage or improve barrier quality [26, 98, 99]. Common outcome measures include time to complete re‐epithelialisation, epithelial migration distance, barrier function metrics such as transepidermal water loss and histologic assessment of epidermal thickness and differentiation. Noninvasive modalities such as laser speckle perfusion imaging, OCT and confocal microscopy can track vascular and epithelial responses in vivo, while the shallow wound depth also facilitates spatially resolved molecular analyses, including microdissection‐based transcriptomic approaches [39, 40, 41].

The translational relevance of this model stems from the strong architectural similarity between porcine and human epidermis and dermis, enabling clinically meaningful prediction of human donor‐site healing. Winter's foundational work in pigs demonstrated that maintaining a moist environment dramatically accelerates re‐epithelialisation, a discovery that transformed clinical practice by motivating the development of modern occlusive dressings [12, 18, 19]. The pig's epidermal and dermal architecture, adnexal density and predominantly epithelial‐driven healing provide greater physiologic relevance than rodent models, in which contraction dominates repair [5, 9, 10]. As a result, the partial‐thickness porcine model remains a preferred platform for assessing early epidermal healing, barrier restoration and performance of dressings and topical therapies [42]. Despite its strengths, the model is limited by its rapid healing trajectory and minimal granulation tissue formation, making it less suitable for evaluating deeper regenerative scaffolds or chronic wound therapies. Meticulous dermatome technique is necessary, as small variations in depth can introduce substantial variability in healing time [40]. Moreover, the model rarely transitions to chronicity without an added perturbation such as ischaemia or repeated injury. Because early regenerated epidermis may lack complete rete ridge architecture, functional restoration of barrier properties should ideally be assessed directly rather than inferred from gross closure alone. Collectively, these considerations highlight the importance of careful depth standardisation, site selection and endpoint choice when using partial‐thickness porcine wounds for translational testing.

### 2.3 Full‐Thickness Excisional Porcine Wound Models

Full‐thickness wounds in pigs are created by excising the entire dermis down to the subcutaneous fat or fascia, producing an open wound that heals by granulation tissue formation, limited contraction and re‐epithelialisation from the wound margins. These excisional wounds, usually circular or square and ranging from 1 to 10 cm in diameter, are placed on the dorsum using a scalpel, trephine or biopsy punch and are left to heal by secondary intention. The model reliably reproduces the principal phases of human full‐thickness wound repair, including fibrin clot formation, leukocyte infiltration, fibroplasia, angiogenesis within granulation tissue and subsequent epithelial migration across the wound bed [5, 10, 14, 31, 43, 44]. Because pigs exhibit limited wound contraction compared with rodents, healing more closely reflects human open‐wound physiology.

Full‐thickness porcine wounds are widely regarded as a predictive preclinical platform for testing wound therapies. In contrast to rodent excisional wounds, which close rapidly by contraction, porcine wounds heal primarily through granulation tissue formation and epithelial migration, producing kinetics and tissue architecture similar to human traumatic wounds, donor‐site failures and chronic wound beds [9, 45]. Moreover, therapeutic outcomes in pig wounds show higher concordance with human clinical results than rodent systems, supporting their translational value for evaluating biomaterials, cell therapies, topical growth factors and systemic agents [14]. The model also permits assessment of long‐term scar formation and remodelling, making it particularly useful when studying fibrotic phenotypes in breeds such as Yorkshire or Red Duroc pigs [46, 47].

Researchers commonly create a grid of 6–10 full‐thickness wounds per animal, spaced to avoid wound merging. Standard wound sizes range from 2 × 2 cm to 10 × 10 cm, large enough to model clinically relevant defects while allowing multiple test conditions per pig [43, 44]. The dermis is completely removed, but whether excision terminates at the subcutaneous fat or extends to the fascia should be reported explicitly, because retained adipose tissue may alter wound‐bed composition and may be particularly relevant in studies of infection or wound‐bed quality. While splints are essential in rodent models to prevent contraction, they are often unnecessary in pigs due to their tightly adherent skin, though some investigators still use stents to improve interstudy consistency [45, 48]. Wound healing is assessed by serial planimetry, perfusion imaging, and histological sampling at defined intervals (e.g., days 3, 7, 14, 21), capturing granulation tissue thickness, vascular density, inflammatory infiltrates, collagen deposition and epithelial migration [44]. Full‐thickness wounds also lend themselves to advanced molecular profiling, including transcriptomics, proteomics and in vivo imaging such as multiphoton microscopy, to characterise extracellular matrix remodelling and angiogenesis in real time [28, 49, 50].

The full‐thickness porcine model has enabled key advances in the development of dermal substitutes, biologic scaffolds and topical growth factor therapies. Numerous studies have shown that engineered matrices, collagen‐based dressings and bilayered skin substitutes enhance granulation quality and accelerate epithelial coverage compared with untreated controls, consistent with subsequent clinical performance in humans [50, 51, 52, 53, 54, 55, 56]. This model has also demonstrated predictable responses to cytokine and growth factor stimulation, including enhanced granulation, angiogenesis and epithelialisation following topical growth factor application, thereby mirroring therapeutic effects later observed in human diabetic ulcer trials [25, 57, 58]. Because full‐thickness wounds generate substantial granulation tissue and eventual scar, they have also been widely used to evaluate fibrosis‐modulating therapies and to differentiate between hypertrophic and normotrophic scar phenotypes, particularly in pig strains with known fibrotic tendencies [66, 67, 68, 69, 89, 90, 91]. Collectively, these features make the model particularly useful for mechanistic studies and product development.

The porcine full‐thickness excisional wound is one of the closest approximations to human dermal injury available in preclinical research. Healing occurs over a time course, often 2–6 weeks depending on wound size and depth, that is sufficient to detect meaningful therapeutic effects. The model supports evaluation of chronic wound mechanisms when combined with overlays such as ischaemia, diabetes or infection, and it generates robust granulation tissue suitable for biochemical, mechanical and morphologic analyses. Importantly, therapeutic responses in this model frequently parallel outcomes observed in human wounds, making it well suited for late‐stage preclinical testing. Although full‐thickness pig wounds heal more slowly than rodent wounds, they still close faster than many chronic human ulcers, which limits their use for modelling nonhealing pathology unless paired with additional impairments. The model requires substantial animal care, repeated monitoring and sometimes sedation for dressing changes or imaging, increasing cost and logistical burden. Pigs also remodel scar tissue more rapidly than humans, meaning that long‐term scar maturation studies must be interpreted cautiously. Nonetheless, for evaluating early and intermediate healing phases, granulation quality and responses to biomaterials or biologics, the model remains highly informative.

### 2.4 Infected Porcine Wound Models

Infected porcine wound models are widely used to study how bacteria impair healing and to evaluate antimicrobial, antibiofilm and debridement‐based therapies in a physiologic environment that closely resembles human wounds. These models generally begin with a partial‐ or full‐thickness excisional wound created under aseptic conditions, after which a defined inoculum of bacteria is deliberately applied to establish either acute infection or a chronic, biofilm‐forming state. The porcine skin microenvironment, adnexal density and immune responses support bacterial colonisation and biofilm development in a manner consistent with human wounds, making pigs a preferred model when testing interventions against clinically relevant pathogens [14, 21, 29].

Human chronic wounds, including diabetic foot, venous leg and pressure ulcers, frequently harbour organisms such as *Staphylococcus aureus* (including MRSA), *Pseudomonas aeruginosa* and polymicrobial consortia that form biofilms and resist host defences [59, 60, 61, 62]. Rodent wounds rarely sustain localised infections that resemble these clinical states, because bacteria are often either cleared rapidly or associated with systemic illness. In contrast, porcine wounds can support stable, localised infection without overt systemic deterioration, allowing sustained evaluation of antimicrobial dressings, antiseptic solutions, topical or systemic antibiotics, debridement strategies and antibiofilm agents [63, 64]. These models also permit repeated sampling of wound tissue and exudate, paralleling the clinical use of wound cultures and biopsies. Notably, porcine wounds reproduce key microbial behaviours observed in human wounds, including biofilm formation, polymicrobial interaction and delayed healing [29, 30, 31].

To establish infection, wounds are inoculated with a known quantity of bacteria (typically 10^6^ to 10^8^ CFU, depending on organism and wound size), often followed by controlled occlusion for 24‐48 h to promote colonization. Single‐species infection models (e.g., MRSA‐only) simulate acute or monomicrobial colonisation, whereas polymicrobial models combining *S. aureus* and *P. aeruginosa* better represent the complex microbial communities observed in chronic human wounds [30, 64, 65]. Biofilm models typically maintain a moist, protected microenvironment to permit biofilm maturation, whereby within 3–5 days infected porcine wounds develop structurally confirmed biofilms containing bacterial aggregates embedded in extracellular polymeric matrix, as demonstrated by microscopy‐based approaches [29, 65]. Outcome measures include quantitative bacteriology, histologic assessment of infection and inflammation, biofilm imaging and standard wound‐healing metrics such as closure rate and epithelial migration. Because multiple wounds are often created on the same animal, careful aseptic techniques and controlled handling during inoculation and dressing changes are essential to minimise unintended cross‐wound contamination. Pigs generally tolerate localised infection without systemic deterioration, but humane monitoring remains essential to detect fever, lethargy or other signs of systemic illness.

Porcine infection models have been used to quantify bacterial burden, visualise biofilm development and relate microbial persistence to delayed closure and inflammation, thereby supporting preclinical testing of antimicrobial and antibiofilm interventions. Early porcine infection studies established that wounds inoculated with *S. aureus* , *P. aeruginosa* or mixed flora can maintain elevated bacterial loads and that topical antiseptics can reduce bacterial burden without impairing epithelialisation [21]. The field advanced substantially when in vivo microscopic documentation of biofilm in mammalian wounds demonstrated that *P. aeruginosa* formed mature biofilms in porcine full‐thickness wounds within several days, accompanied by delayed healing and persistent inflammation [29]. Polymicrobial infection studies further showed that combined MRSA and *P. aeruginosa* infection produced more extensive biofilm, stronger inflammatory responses and slower closure than single‐species infections [30]. Porcine models have also been used to evaluate antimicrobial technologies; for example, nanocrystalline silver dressings reduced bacterial load and modulated matrix metalloproteinases in infected porcine wounds [100]. Collectively, these findings demonstrate how infected porcine wound models clarify the impact of infection on healing and support development of antimicrobial and antibiofilm therapies (Table 2).

**TABLE 2:** Emerging and specialised porcine wound model approaches.

Approach	Induction strategy/defining concept	Key applications	Representative references
Chronic‐like glutaraldehyde‐based model	Burn‐excision/full‐thickness wound platform with topical glutaraldehyde to induce delayed healing, matrix disruption and persistent inflammation.	Chronic‐like impaired healing; matrix remodelling defects; comparative transcriptomic/lipidomic studies relevant to VLU/DFU‐like biology.	[32]
Resiquimod‐induced hyperinflammatory wound model	Topical resiquimod (TLR7/8 agonist) applied to full‐thickness porcine wounds to induce prolonged local inflammation and delayed healing.	Hyperinflammatory impaired‐healing studies; mechanistic investigation of inflammation‐driven delay in repair; complementary chronic‐like platform distinct from ischemic, diabetic or infected models.	[94, 101, 102]
Radiation dermatitis/irradiated wound model	Controlled local irradiation of porcine skin, with or without subsequent wounding in previously irradiated fields.	Dose–response studies, radiodermatitis mitigation, radiation‐impaired repair, fibrosis and regenerative therapy testing.	[103, 104, 105, 106, 107]
Accelerated‐aging/photoaging‐related approaches	Repeated UV exposure to induce photoaging‐like changes; conceptually distinct from intrinsic ageing.	Exploratory studies of altered skin structure and potential age‐associated changes in wound response.	[108]
Progeroid/genetically accelerated ageing swine	Gene‐edited progeroid pigs (e.g., LMNA‐mutant models) exhibit premature ageing phenotypes.	Emerging platform for studying systemic ageing biology; wound‐healing applications remain incompletely characterised.	[109]
Humanised/immunologically modified pigs	Immunodeficient or genetically modified swine engrafted with human cells or designed to better support human immune biology.	Human‐specific immune responses, cell therapy testing and translational modelling not feasible in conventional porcine systems.	[110, 111, 112]
CRISPR/genetically engineered porcine systems	Targeted gene editing in pathways related to metabolism, immunity, or tissue repair.	Mechanistic disease modelling and hypothesis‐driven preclinical studies in a large‐animal context.	[109, 110, 111, 112]
Spatial omics‐enhanced wound studies	Spatial transcriptomics/proteomics and related molecular mapping layered onto established porcine wound models.	Compartment‐resolved biology, cell‐state localisation, pathway mapping and human–pig comparative analyses	[33]
AI‐enabled imaging/computational analytics	Integration of serial wound imaging, morphometrics and multi‐omics to develop predictive or classification frameworks	Healing‐trajectory classification, biomarker discovery and computationally enhanced outcome assessment	[33, 113]

Infected porcine wounds reproduce key features of human infected wounds, including biofilm formation, polymicrobial interaction, inflammatory changes and associated delays in healing. The model accommodates both mono‐ and polymicrobial infection, provides sufficient tissue for repeated sampling and enables controlled testing of topical agents, systemic antibiotics, antibiofilm compounds and debridement methods. Establishing consistent infection requires careful titration of inoculum and controlled wound conditions, because bacterial doses that are too low may be cleared whereas excessive inoculation may increase the risk of systemic spread. Variability between wounds on the same animal can also occur because of local microenvironmental differences. Although porcine biofilms form robustly, the duration of infection is shorter than in many human chronic ulcers, and chronic‐like infection models may therefore require repeated inoculation or additional impairments such as ischaemia or diabetes. Large‐animal infection studies also require appropriate biosafety practices and intensive monitoring. Despite these constraints, infected porcine wound models remain among the most clinically relevant platforms for studying bacterial pathogenesis, biofilm biology and antimicrobial intervention performance [29, 59, 60].

### 2.5 Burn Wound Models

Thermal injury models in pigs involve inducing burns of defined depth and evaluating both acute injury responses and subsequent healing or scarring. Pigs are a preferred burn model because their skin responds to thermal injury with blistering, dermal necrosis, inflammation, fluid loss and scarring patterns that closely resemble those seen in humans [14, 114]. Burn models may be created as partial‐thickness injuries to study burn wound conversion, infection and epithelial repair or as full‐thickness burns to investigate excision strategies, grafting techniques and scar formation [26, 115]. Porcine burn models have played a central role in advancing modern burn care, supporting the development of topical antimicrobials, early excision strategies and skin substitute technologies [114, 115, 116, 117, 118]. Partial‐thickness porcine burns reliably demonstrate a zone of stasis, defined as marginally perfused tissue that may either recover or progress to necrosis, a concept that has been extensively characterised using porcine comb‐burn models [119, 120]. Deep dermal burns in pigs frequently progress to hypertrophic scarring, particularly in breeds such as the Red Duroc, making pigs an important model for studying anti‐scarring interventions and fibroproliferative signalling pathways [26, 92, 93]. Pigs also mount systemic inflammatory and metabolic responses to larger burns that approximate those observed in human patients, enabling investigation of both local and systemic burn pathophysiology [114].

Burns in pigs are produced using standardised thermal devices to ensure reproducible depth and size. Contact burns are most commonly used and involve applying a heated metal device or hot water‐filled applicator to the skin for a defined duration to achieve controlled injury depth [26]. Scald burns are used less frequently and may produce more variable injuries, while flame, electrical and chemical burns are less commonly employed because of challenges in standardisation [114, 116]. The comb burn model produces alternating zones of full‐ and partial‐thickness injury in a single wound, allowing direct study of burn wound conversion and interventions aimed at preserving the zone of stasis [119]. Burns may be allowed to heal spontaneously or excised at defined time points to study early versus delayed surgical management [121]. Outcomes commonly assessed include histologic depth of injury, time to epithelial closure or graft take, infection rates and scar characteristics such as thickness, erythema and collagen organisation [26, 93]. Burn wounds are also frequently used to evaluate topical antimicrobials and dressings, including in settings where controlled bacterial inoculation is applied to assess infection control [23, 122, 123, 124, 125].

In addition to phenotypic differences, recent transcriptomic analyses have provided mechanistic insight into breed‐specific healing responses in porcine burn models. Longitudinal RNA sequencing studies comparing red Duroc and Yorkshire pigs in deep partial‐thickness burn wounds showed that red Duroc wounds exhibited a more robust and prolonged inflammatory immune response, increased fibroblast migration and proliferation and enhanced extracellular matrix modulation relative to Yorkshire wounds [126]. Collectively, the red Duroc deep partial‐thickness burn wounds produced a greater hypertrophic scar‐like response than Yorkshire deep partial‐thickness wounds, underscoring the importance of breed selection in porcine burn studies and suggesting that underlying gene‐expression programmes contribute to variability in healing and scarring outcomes.

Recent porcine burn studies have also expanded into cell‐based regenerative approaches. In a full‐thickness burn‐excised wound model, umbilical cord mesenchymal stromal/stem cells delivered on a dermal regeneration template were associated with improved epithelialisation, vascularisation and scar‐related outcomes, with lower cell doses showing favourable effects in this setting [127]. More recently, cord tissue‐derived induced pluripotent stem cell‐derived mesenchymal stem cells incorporated into Integra in a porcine full‐thickness burn model were likewise reported to improve healing‐associated outcomes, including epithelial recovery and histologic features of tissue restoration [128]. These studies further support the value of porcine burn models for preclinical evaluation of regenerative cell‐based therapies.

A major strength of the porcine burn model is its close resemblance to human burn biology, including blister formation, burn wound progression and the potential for hypertrophic scarring [14, 26, 116]. The model has been instrumental in optimising skin substitutes and surgical strategies that were subsequently translated into clinical practice [56, 117]. Pigs also permit serial biopsies and longitudinal assessment of burn healing, which is rarely feasible in human patients. However, several limitations should be considered. Burn depth uniformity requires precise control of temperature, contact time and pressure during injury induction and variations in subcutaneous fat thickness can influence heat conduction and injury severity [116]. Large‐area burns can induce systemic effects requiring intensive supportive care, so many studies limit total body surface area involvement. Additional considerations include logistical complexity, cost and ethical requirements related to pain management and animal welfare. Despite these challenges, porcine burn models remain one of the most clinically relevant platforms for studying burn injury, healing and scarring. Importantly, burn wound models can also serve as a platform for studying impaired or chronic‐like healing when combined with additional perturbations such as infection, ischaemia or metabolic dysfunction, thereby providing a conceptual bridge to chronic wound modelling approaches discussed in subsequent sections.

## 3 Moving Toward Chronic‐Like Porcine Wound Models

### 3.1 Diabetic Porcine Models

Diabetes impairs wound healing through multiple mechanisms, including dysregulated inflammatory responses and altered growth factor signalling. Human diabetic foot ulcers typically arise from a combination of neuropathy, impaired perfusion and intrinsic cellular dysfunction. Rodent diabetic models exhibit some healing delay but still depend heavily on wound contraction and are limited by small wound size and differing skin architecture [5, 9, 11]. In contrast, diabetic pigs develop wound phenotypes that more closely resemble human diabetic ulcers, including prolonged inflammation, impaired granulation tissue formation, reduced angiogenesis and increased susceptibility to infection [27, 31, 75]. These features make diabetic pigs useful for evaluating biologic, biomaterial and vascular or metabolic interventions intended to improve impaired wound healing.

Diabetic porcine models are used to investigate how hyperglycaemia and metabolic dysfunction impair wound healing, mirroring key clinical features of diabetic foot ulcers [27, 31]. The most common approach is chemical induction using STZ, which selectively destroys pancreatic β‐cells and produces a sustained hyperglycaemic state [76, 77]. Alternatively, diet‐induced or genetically predisposed miniature pigs, such as Ossabaw or Yucatan strains, can develop metabolic syndrome characterised by insulin resistance, dyslipidaemia and obesity [78, 79]. Once diabetes is established, investigators typically create standardised full‐thickness excisional wounds to assess delays in inflammation resolution, granulation tissue formation, angiogenesis and epithelialisation, all of which are hallmarks of impaired healing in diabetic patients.

STZ‐induced diabetes in Yorkshire pigs typically develops within days of administration, resulting in persistent hyperglycaemia (> 250‐300 mg/dL) unless partially corrected with insulin. Researchers commonly maintain pigs in a moderate hyperglycaemic range to model poorly controlled human diabetes while avoiding ketoacidosis [27]. Once stabilised, standardised full‐thickness excisional wounds, often 2‐3 cm in diameter, are created and healing is monitored over several weeks. Diabetic pig wounds consistently heal more slowly than non‐diabetic controls, with delayed epithelialisation and granulation tissue formation, prolonged inflammation and diminished angiogenesis [27, 77]. Diet‐induced or metabolically challenged minipigs offer additional translational relevance by incorporating obesity, insulin resistance and dyslipidaemia, all of which are common comorbidities in human diabetic wounds [79]. Experimental endpoints often include wound closure kinetics, granulation tissue thickness, microvascular density, fibroblast proliferation and collagen deposition [80]. While neuropathy does not automatically occur in short‐term STZ models, simulated neuropathic impairment can be introduced through local denervation to assess its contribution to delayed healing [70].

Diabetic porcine wound models have been instrumental in demonstrating how hyperglycaemia impairs multiple phases of wound repair. Studies consistently show that diabetic pig wounds exhibit slower granulation tissue formation, blunted angiogenic responses and reduced tensile strength compared with non‐diabetic wounds [27, 31]. These models have also been used to evaluate therapies intended to improve diabetic wound healing, including growth factor‐based approaches, dermal substitutes and metabolically or vascularly targeted interventions [71, 72, 73, 80]. When combined with infection overlays, diabetic pigs exhibit increased bacterial burden and further delayed closure, paralleling features of infected diabetic ulcers in the clinic.

The primary strength of diabetic swine models lies in the combination of a diabetic systemic milieu with a human‐scale wound environment, enabling clinically relevant testing of dressings, biologics and other interventions. The model supports realistic wound sizes, serial biopsies, imaging and use of full‐scale clinical dressings and devices. Type II‐like diabetic minipigs provide an additional layer of clinical relevance by incorporating obesity, insulin resistance and metabolic dysfunction. However, several limitations should be considered. Diabetes induction and maintenance can be technically demanding, and short‐duration STZ models may not fully reproduce long‐term complications such as neuropathy or advanced vasculopathy unless these are specifically incorporated into the study design. In addition, even diet‐induced diabetic porcine models do not fully replicate the cumulative tissue injury that typically precedes diabetic foot ulcer development in humans. Thus, although diabetic porcine models are highly informative for studying impaired healing and evaluating candidate therapies, they remain a partial representation of the chronic human disease state.

### 3.2 Ischaemic Wound Models

Ischaemia, defined as inadequate tissue perfusion and oxygen delivery, disrupts wound repair by impairing cellular metabolism, inflammatory cell recruitment, angiogenesis and extracellular matrix deposition, resulting in delayed granulation tissue formation and increased risk of tissue necrosis [14, 48]. Chronic ischaemia is a key contributor to many non‐healing human wounds, including arterial insufficiency ulcers and a substantial subset of diabetic foot ulcers associated with peripheral arterial disease (PAD) [74]. Although rodent ischaemia models have provided mechanistic insight, their translational utility is limited by small wound size, rapid closure driven by contraction and differences in cutaneous vascular architecture [45]. In contrast, porcine models permit creation of localised, sustained reductions in perfusion without systemic instability, enabling controlled study of hypoxia‐driven impairment in human‐scale wounds. Porcine skin closely resembles human skin in thickness, dermal structure and microvascular organisation, allowing reproducible induction of regional perfusion deficits while preserving tissue viability for longitudinal assessment [81].

The most widely used porcine ischaemic wound model is the bipedicle dorsal skin flap model [28]. In this approach, a rectangular section of dorsal skin is surgically elevated and undermined, leaving it attached only at its cranial and caudal pedicles. Disruption of intervening perforating vessels reduces blood flow, particularly in the central region of the flap. After a stabilisation period of approximately 24‐48 h, standardised full‐thickness excisional wounds are created within the ischaemic zone and compared with identical wounds placed in adjacent, normally perfused skin in the same animal [28]. This model produces a measurable reduction in perfusion, typically quantified using techniques such as laser Doppler flowmetry or transcutaneous oxygen measurements [28]. Common experimental endpoints include wound closure kinetics, granulation tissue thickness, capillary density, inflammatory cell profiles, expression of hypoxia‐responsive markers such as HIF‐1α and tensile strength of healed tissue. The model is compatible with clinically relevant perfusion‐monitoring approaches, enabling correlation between perfusion deficits and healing outcomes [82, 83].

Using the bipedicle flap model, ischaemic wounds exhibit delayed closure, reduced early inflammatory cell infiltration, diminished granulation tissue formation, impaired angiogenesis and altered remodelling compared with normally perfused wounds [28]. Gene expression profiling has shown upregulation of hypoxia‐ and stress‐associated pathways together with downregulation of proliferative and matrix synthesis signals [28], features that parallel those reported in human ischaemic ulcers [48, 74]. This model has also been used to evaluate therapies aimed at improving perfusion or mitigating hypoxia‐driven dysfunction. Pro‐angiogenic interventions, including VEGF‐based strategies, have been associated with improved neovascularisation and earlier healing responses in ischaemic porcine wounds [28, 74]. Hyperbaric oxygen therapy has likewise been examined and can transiently improve tissue oxygenation and early healing responses; however, these effects do not eliminate the underlying perfusion deficit, consistent with clinical evidence that oxygen therapy is supportive rather than substitutive for revascularisation in severe ischaemia [129]. Earlier porcine studies using alternative ischaemic paradigms, such as combined compression and denervation, similarly demonstrated that inadequate perfusion alone is sufficient to stall wound repair, reinforcing the central role of ischaemia in chronic wound pathophysiology.

The ischaemic porcine wound model reproduces key aspects of arterial insufficiency, including healing within a persistently hypoxic, poorly perfused environment. Key strengths include reproducible and quantifiable perfusion deficits, internal controls within the same animal, compatibility with clinically used perfusion‐imaging techniques and suitability for testing pro‐angiogenic, vascular‐targeted and oxygen‐based therapies. The ability to link perfusion metrics directly to histologic and functional healing endpoints substantially enhances translational relevance [14, 28, 48, 74, 83]. However, several limitations should be considered. Flap creation is invasive and technically demanding, and excessive disruption of the pedicles can lead to necrosis rather than stable ischaemia. Variability in flap geometry and vascular anatomy also necessitates careful standardisation to achieve consistent perfusion deficits. In addition, the model primarily represents acute surgically induced ischaemia, whereas many human chronic wounds arise from long‐standing vascular disease with partial collateralisation [28]. The model also reflects arterial insufficiency but does not reproduce venous hypertension, which underlies venous ulcer pathology. Despite these limitations, the ischaemic porcine wound model remains a highly useful platform for studying perfusion‐limited wound healing and evaluating vascular‐directed therapies.

### 3.3 Pressure Ulcer Models

Pressure ulcers (pressure injuries) are localised wounds caused by sustained mechanical loading over bony prominences, leading to prolonged ischaemia, tissue hypoxia and reperfusion injury that culminate in progressive necrosis of skin and underlying tissues. Clinically, they represent a major problem in immobilised or neurologically impaired patients and often originate in deep muscle layers before superficial skin breakdown becomes evident, a phenomenon known as deep tissue injury [84, 85]. Rodent models have provided some mechanistic insight, but their small tissue volume, differences in skin anatomy and difficulty in reproducing human‐scale pressure distributions limit translational relevance [4, 31]. IBy contrast, porcine models more closely resemble human skin structure and allow application of controlled pressure over clinically relevant surface areas. This enables systematic study of pressure magnitude, exposure duration and microclimate as determinants of ulcer formation and progression [4, 31].

The most extensively characterised porcine model was developed by Kokate and colleagues [85]. In this model, defined pressures, typically in the range of ~50‐100 mmHg, are applied for controlled durations under regulated temperature and humidity conditions. These studies produced a spectrum of injuries ranging from transient erythema to full‐thickness ulcers and showed that deep muscle necrosis can precede visible skin breakdown, consistent with clinical deep tissue injury [85]. They also established that tissue damage depends on both pressure magnitude and exposure duration, whereby lower pressures sustained over longer periods can result in irreversible injury, whereas higher pressures applied briefly may not [86]. In addition, they demonstrated that a warm, moist microclimate exacerbates injury severity, supporting the concept that temperature and moisture are important modifiers of pressure ulcer susceptibility [85]. Subsequent porcine studies have reinforced these mechanistic insights. Experiments incorporating cycles of pressure‐induced ischaemia followed by reperfusion showed that restoration of blood flow can exacerbate tissue injury through oxidative stress mechanisms. In these models, significant muscle necrosis occurred after reperfusion rather than during the ischaemic interval alone, and antioxidant interventions reduced this injury, implicating reactive oxygen species in pressure ulcer pathogenesis [87]. Collectively, these findings support the view that pressure ulcers arise from a combination of sustained mechanical loading, ischaemia and reperfusion‐associated injury rather than superficial trauma alone.

Porcine pressure ulcer models have contributed to the development and evaluation of preventive and therapeutic strategies. Because loading conditions can be precisely controlled, these models have been used to test interventions such as pressure‐redistributing surfaces, positioning strategies and microclimate modification under conditions that approximate human care [85]. Observations from porcine models, including the importance of early off‐loading and the recognition that clinically subtle skin changes may overlie deep tissue damage, have informed approaches to prevention and risk assessment. In addition, these models have been used to evaluate advanced dressings, biomaterials and device‐based therapies prior to clinical translation. Together, these studies support the use of porcine pressure ulcer models as a practical preclinical system for preventive and therapeutic testing.

Porcine pressure ulcer models offer high reproducibility and strong translational relevance. They reliably generate deep tissue injury analogous to human pressure ulcers, allowing investigators to follow ulcer development and evaluate interventions under controlled conditions [86]. The ability to impose human‐scale pressures and observe tissue responses over time is a major strength of the model. However, several limitations should be considered. These models typically require anaesthesia or restraint, which may influence perfusion and healing, and ethical constraints often limit study duration, making late chronic phases less accessible. There is also some anatomical mismatch, as pressure is commonly applied to porcine flanks or backs rather than to human predilection sites such as the sacrum or heel. Nonetheless, the fundamental biomechanical and biologic principles derived from these models, including pressure–time injury thresholds, deep tissue vulnerability and the roles of microclimate and reperfusion, have broad relevance to human disease. Overall, porcine pressure ulcer models remain highly informative systems for studying pathophysiology and evaluating preventive and therapeutic strategies [85, 86, 87, 88].

## 4 Emerging and Specialised Porcine Wound Model Approaches

### 4.1 Chronic‐Like Porcine Models and Glutaraldehyde‐Induced Wound Model

Chronic wounds in humans are defined by failure to progress through the normal phases of healing and are typically associated with multifactorial pathophysiology, including vascular insufficiency, metabolic dysfunction, infection and age‐related impairment. In contrast, experimental wounds in pigs, even when large or deep, generally follow an orderly healing trajectory and close within weeks. As a result, the development of true chronic wound models in porcine systems remains challenging. Most porcine approaches described as “*chronic wound models*” are therefore more accurately characterised as chronic‐like or impaired‐healing systems, in which specific perturbations are introduced to delay or stall repair and thereby reproduce selected aspects of human chronic wound biology rather than the full clinical phenotype (Table 2). Several strategies have been used to generate chronic‐like wound environments in pigs. These include systemic or local impairments such as diabetes, ischaemia, infection, pressure‐induced injury, radiation exposure and chemically mediated perturbations. Increasing attention has also been directed toward combining multiple impairments within a single model, since diabetic wounds with superimposed ischaemia or infection often exhibit more pronounced delays in healing than single‐perturbation systems. Such combined approaches more closely approximate the multifactorial nature of human chronic wounds, although they remain experimentally constructed models and do not fully reproduce the long‐term disease progression observed clinically.

Among emerging approaches to induce chronic‐like wound states in pigs, a chemically induced paradigm using topical glutaraldehyde (GA) has gained attention [32]. In this model, standardised thermal injuries are first created using heated brass rods and are then debrided/excised with a trephine to generate full‐thickness wounds. The initial burn is used to create an early zone of ischaemia localised primarily at the wound edge, after which topical GA is applied to induce localised tissue injury, matrix disruption and delayed healing responses. Excision of the burn eschar and devitalised tissue allows clearer assessment of the subsequent healing dynamics and of the effects of GA crosslinking in the remaining wound bed. This burn‐excision, full‐thickness wound platform is therefore used not as a burn model per se, but as the substrate for a chronic‐like impaired‐healing system. The resulting phenotype has been described as including delayed epithelialisation, persistent inflammation, extracellular matrix disruption, altered collagen ultrastructure and impaired healing progression. Transcriptomic and lipidomic analyses have also been used to compare GA‐treated wounds with human venous leg ulcers and diabetic foot ulcers, identifying similarities in selected signalling pathways and metabolite profiles [32].

A related but mechanistically distinct strategy is the induction of prolonged local hyperinflammation using resiquimod, a Toll‐like receptor 7/8 agonist. In a porcine full‐thickness excisional wound model, topical resiquimod applied daily for 6 days induced visible wound inflammation and delayed healing relative to untreated control wounds [101, 102]. Histologic and molecular analyses showed increased immune‐cell infiltration together with increased expression of inflammatory and remodelling‐associated markers including IL6, MMP1 and CD68, supporting the concept that exaggerated inflammatory signalling alone can shift an otherwise normally healing porcine wound toward a more chronic‐like state. This resiquimod‐based paradigm is therefore a useful complementary platform for studying hyperinflammatory impaired healing, particularly in settings where persistent inflammation is considered a major driver of chronic wound pathophysiology rather than ischaemia, metabolic dysfunction or infection alone [101, 102].

The relevance of inflammation‐driven chronic‐like porcine models is further supported by subsequent work in Duroc pigs, in which resiquimod‐induced hyperinflammation in full‐thickness and burn wounds was associated with more pronounced hypertrophic scar features than standard full‐thickness wounds. In that study, resiquimod‐induced wounds showed higher inflammatory‐cell infiltration, relative hypoxia and scar characteristics resembling human hypertrophic scars, suggesting that persistent inflammation in porcine wounds may influence not only delayed closure but also downstream fibroproliferative remodelling. Although this later application is more directly relevant to scar biology than to chronic ulceration, it further highlights the versatility of resiquimod‐based inflammatory induction as a tool for interrogating pathologic wound‐healing environments [94].

A central strength of these chronic‐like porcine approaches is their ability to induce localised impairment of healing in a species that otherwise exhibits robust repair, while still allowing within‐animal comparisons across multiple wounds and treatment conditions. These models have also been reported to show reduced responsiveness to standard wound treatments and improvement following interventions such as debridement, findings that are broadly consistent with impaired‐healing states. However, several limitations should be considered. Both the GA and resiquimod models represent experimentally imposed perturbations of wound biology rather than spontaneous or systemic chronic wound disease states and therefore do not inherently reproduce the full complexity of human chronic wounds. In addition, careful optimisation of exposure conditions is required to avoid excessive tissue injury or inflammatory exaggeration that may limit interpretability. As relatively recent approaches, broader validation, standardisation across laboratories and direct comparison with other chronic‐like porcine systems will be necessary to define their place within the preclinical pipeline. Overall, chronic‐like porcine wound models, including GA‐based and resiquimod‐induced paradigms, are best viewed as complementary systems that reproduce selected aspects of impaired healing rather than complete representations of human chronic wound pathology.

### 4.2 Hypertrophic Scar Models

Hypertrophic scars are excessive fibroproliferative scars that develop after deep dermal injury and are characterised by persistent myofibroblast activity, elevated TGF‐β signalling and dense collagen deposition. Clinically, they present as raised, erythematous lesions that may cause contractures, discomfort or disfigurement and effective preventive therapies remain limited [95]. Small‐animal models do not reproduce hypertrophic scarring well, because rodent wounds heal predominantly by contraction with relatively limited fibrotic response, and murine scars lack the characteristic collagen architecture of human hypertrophic scars [8]. By contrast, porcine models, particularly specific breeds, can reproduce many features of human hypertrophic scarring [96]. Pigs have skin anatomy and wound‐healing physiology that closely resemble humans, and breeds such as the Red Duroc have a predisposition to fibroproliferative healing, making them valuable for hypertrophic scar research [89, 97]. In the Red Duroc model, deep dermal injuries produce raised scars over 2‐3 months that resemble human hypertrophic scars macroscopically and microscopically [93]. Deep partial‐thickness wounds in female Red Duroc pigs form thick scars by 8‐12 weeks, whereas comparable wounds in Yorkshire pigs yield flatter scars. These scars exhibit disorganised collagen bundles, persistent α‐smooth muscle actin (α‐SMA)‐positive myofibroblasts and increased expression of profibrotic mediators such as TGF‐β1 and IGF‐1 compared with uninjured skin [46, 89, 93, 97]. Red Duroc wounds also show prolonged elevation of TGF‐β1 and IGF‐1 together with reduced decorin expression, a molecular pattern that parallels features reported in human hypertrophic scars [93]. Genetic studies further support a heritable contribution, as cross‐breeding Red Duroc and Yorkshire pigs produced offspring with intermediate scar phenotypes [46].

Porcine hypertrophic scar models also enable investigation of scar pathophysiology and preclinical evaluation of anti‐scarring therapies in a setting that resembles human wound healing. These models have been used to study mechanisms such as sustained inflammation in scar formation. In one study, prolonged local inflammation induced in Duroc pig wounds by repeated TLR7/8 agonist application generated standardised hypertrophic scars, supporting a role for persistent inflammation in more severe scar formation [94]. Scar outcomes in pigs can be assessed using clinically analogous measures, including scar height or thickness, pigmentation or redness, pliability and histologic or molecular analyses of collagen organisation, myofibroblast persistence and expression of fibrosis‐related mediators. Red Duroc scars also express several biomarkers associated with human hypertrophic scars, including increased collagen I/III, tenascin‐C, proteoglycans and altered vascular and mast cell patterns [96]. This model has therefore been widely used for preclinical therapeutic testing. For example, cell‐based therapy has been investigated in deep partial‐thickness wounds in Red Duroc pigs and was associated with reduced scar stiffness and hyperpigmentation together with more organised collagen architecture [130]. Laser‐based approaches have also been evaluated, with ablative fractional CO_2_ and Er:YAG treatment improving scar appearance and remodelling parameters in Duroc hypertrophic scars [92]. In addition, interventions already used clinically, such as silicone gel sheeting and intralesional corticosteroids, have shown measurable improvements in porcine scars [96]. Collectively, these studies support the utility of the porcine hypertrophic scar model as a translational platform for mechanistic investigation and therapy development.

The porcine hypertrophic scar model offers several important strengths. It reproduces many morphologic and biologic features of human hypertrophic scarring, enabling mechanistic study of processes such as TGF‐β signalling, prolonged inflammation and injury depth under in vivo conditions. A major advantage is the reproducibility of fibroproliferative scarring in Red Duroc pigs after appropriately calibrated deep dermal injury, which allows controlled comparison of interventions over a clinically relevant time course. Serial biopsies at multiple time points further support detailed analysis of scar maturation and molecular remodelling. Pig studies have also contributed to clinical understanding of injury‐depth dependence in hypertrophic scar formation [131]. However, several limitations should be considered. Large‐animal scar studies are expensive, logistically demanding and require prolonged follow‐up to allow scar maturation. Access to Red Duroc pigs may be limited, and scar characteristics can vary by breed; for example, Guangxi Bama minipigs also form hypertrophic scars but with some differences in phenotype [132]. Precise control of injury depth is essential, as wounds that are too shallow may fail to form hypertrophic scars, whereas excessively deep wounds may heal differently [97]. In addition, porcine models do not reproduce true keloids, and objective scar metrics cannot fully capture subjective symptoms such as pain or pruritus. Despite these limitations, porcine hypertrophic scar models remain among the most informative preclinical systems for studying fibroproliferative scarring and evaluating anti‐scarring interventions.

### 4.3 Radiation Dermatitis Models

Radiation therapy to the skin can result in both acute and chronic injury. In porcine radiation dermatitis models, defined areas of skin are exposed to controlled doses of ionising radiation to induce injury ranging from mild erythema to chronic ulceration, depending on dose. These models are used to study radiation‐induced skin damage and evaluate candidate interventions. Following irradiation, tissue responses can be monitored over time, with lower doses typically producing transient epidermal injury and higher doses associated with more severe tissue damage and delayed healing. Animals are often followed over weeks to months to assess both acute and late effects, including fibrosis and dermal structural change. Common endpoints include dermatitis severity scoring, time to re‐epithelialisation and histologic evaluation of epidermal and dermal injury.

In porcine models, graded doses of ionising radiation produce dose‐dependent skin injury that parallels key features of human radiodermatitis [103]. Single‐fraction electron beam exposure to dorsal skin fields results in a spectrum of responses ranging from transient erythema and desquamation at lower doses to ulceration and dermal necrosis at higher doses [103]. These dose‐dependent effects have been characterised by clinical observation, histologic analysis and molecular assessment, including loss of adnexal structures, microvascular injury and increased expression of inflammatory and fibrotic mediators after higher‐dose exposure. Such findings support the use of porcine models to study dose–response relationships relevant to radiation‐induced skin injury. In addition, full‐thickness wounds created in previously irradiated porcine skin exhibit delayed healing, impaired granulation and reduced angiogenesis compared with non‐irradiated controls, reflecting important features of radiation‐impaired repair [104].

Porcine radiation dermatitis models have also been used to evaluate candidate interventions under controlled conditions. Studies in mini‐pigs have reported that topical Vitamin B_12_ ointment accelerated re‐epithelialisation and reduced inflammatory and fibrotic markers after high‐dose radiation exposure [105]. Other studies found that topical curcumin reduced the severity of acute radiodermatitis and helped preserve epidermal structure after irradiation [106]. Growth‐factor‐based approaches, including keratinocyte growth factor, have also shown protective effects on epithelial tissues in preclinical settings [107]. Together, these findings highlight the utility of porcine models for testing therapies targeting radiation‐induced skin injury, although broader validation remains necessary before extrapolating to clinical practice.

Porcine radiation dermatitis models offer several advantages, including structural similarity between porcine and human skin and the ability to generate reproducible, dose‐dependent injury. These models enable controlled investigation of radiation‐induced skin damage across a range of severities and support longitudinal assessment of healing responses. However, several limitations should be considered. Differences in radiosensitivity between species necessitate careful dose calibration, and healing kinetics in pigs may differ from those observed in human patients. In addition, chronic radiation injury in pigs may partially resolve over time, whereas comparable human wounds may persist longer or require intervention. Practical considerations, including the need for specialised irradiation facilities and extended monitoring, also limit widespread use. Despite these constraints, porcine models provide a valuable experimental platform for studying radiation‐induced skin injury and evaluating candidate therapies.

### 4.4 Accelerated Ageing Wound Models

Ageing has a well‐established impact on wound healing, and chronic wounds predominantly occur in older populations. However, the long lifespan of domestic pigs limits the feasibility of using naturally aged animals for experimental studies. As a result, accelerated‐ageing approaches have been explored. One such method is ultraviolet (UV)‐induced photoaging, in which repeated sub‐erythematogenic UV exposure is used to induce features associated with chronic sun damage. Although porcine skin is frequently used in ex vivo photoaging studies, in vivo models remain limited, with relatively few published reports [108]. In available studies, photoaged porcine skin demonstrates structural and histologic changes, including alterations in dermal organisation, increased vascularity and features consistent with elastosis [108]. However, the effects of these changes on wound‐healing outcomes have not been systematically evaluated. Importantly, photoaging does not replicate intrinsic ageing. Chronic wounds in humans commonly arise in anatomical locations with limited UV exposure, and photodamage introduces features such as solar elastosis and UV‐associated DNA damage that are distinct from age‐related degeneration. Consequently, the relevance of photoaged porcine skin as a model of age‐associated impaired wound healing remains uncertain and requires further validation.

An alternative strategy to model accelerated ageing involves the use of progeroid animals, a concept previously applied in rodent systems. A porcine progeroid model has been generated using CRISPR‐Cas9 gene editing to introduce a heterozygous LMNA mutation (c.1824C > T) associated with Hutchinson‐Gilford progeria syndrome [109]. This model exhibits features of premature ageing and early mortality. While cutaneous and hair abnormalities have been reported, its application to wound‐healing research remains limited. To date, this model has not been extensively characterised in the context of cutaneous wound repair, and its availability is restricted. As a result, its utility for studying age‐related impairment in wound healing in large‐animal systems is not yet established. Together, these approaches highlight the ongoing challenge of modelling intrinsic ageing in porcine systems and underscore the need for models that more closely integrate systemic and tissue‐specific features of age‐associated wound impairment.

## 5 Strengths and Limitations of Porcine Wound Models

Building on the range of acute wound paradigms (Section 2), chronic and comorbidity‐driven models (Section 3) and emerging or specialised approaches (Section 4), it is important to evaluate the overarching strengths and limitations of porcine wound models to better contextualise their translational utility.

### 5.1 Strengths

Porcine skin shares several key structural and functional features with human skin, including comparable epidermal thickness, dermal collagen organisation and capacity to support large, standardised wounds suitable for device‐scale testing and serial sampling (Figure 1). Pigs exhibit similar epidermal thickness ranges (~30‐140 μm) and dermal architecture, including well‐developed rete ridges and a tightly adherent dermis, resulting in wound closure primarily through re‐epithelialisation and granulation tissue formation rather than contraction. As a consequence, the sequence of wound healing in pigs (haemostasis, inflammation, proliferation and remodelling) closely parallels that observed in humans, supporting the use of porcine systems as translational platforms for studying cutaneous repair.

A major strength of porcine wound models is their predictive validity, as experimental outcomes frequently show higher concordance with human clinical responses compared with small‐animal models. Foundational observations in pigs informed the development of core wound‐care practices, including moist wound healing and NPWT, highlighting their value in bridging mechanistic studies and clinical application. In addition, the ability to generate wounds of clinically relevant size allows testing of full‐scale medical devices, biomaterials and dressings under conditions that approximate human use.

The size and surface area of pigs also provide important experimental advantages. Multiple standardised wounds can be created on a single animal, enabling within‐animal comparisons and reducing inter‐subject variability. This design increases statistical efficiency and allows parallel testing of multiple treatments under controlled conditions. Furthermore, porcine models support longitudinal assessment through serial biopsies and noninvasive imaging modalities such as ultrasound, laser Doppler and optical coherence tomography, facilitating detailed temporal analysis of wound‐healing dynamics that is difficult to achieve in smaller animal systems.

Porcine wound models are also highly compatible with advanced analytical approaches. Integration of transcriptomic, proteomic, lipidomic and imaging‐based datasets enables linking of histologic and clinical endpoints with underlying molecular programs. These capabilities support increasingly sophisticated analyses of wound progression and therapeutic response and may facilitate development of predictive frameworks when combined with well‐annotated datasets. Collectively, these features make porcine models well suited for late‐stage preclinical evaluation of therapies and devices under conditions that closely approximate clinical practice (Table 3). Importantly, these strengths are particularly relevant when porcine systems are used to model complex or impaired‐healing states, where large‐animal physiology and clinically relevant wound scale provide advantages over reductionist systems.

**TABLE 3:** Strengths and limitations of porcine wound models.

Strengths	Limitations
Anatomical/physiological similarity: Porcine skin closely mimics human epidermal thickness, dermal structure, adnexal glands	High cost and resource needs: Housing, anaesthesia and care for large animals are expensive and labour intensive
High predictive validity: Wound healing outcomes in pigs correlate ~78% with human clinical results (vs ~53% for rodent models)	Ethical considerations: Use of intelligent, sentient animals demands rigorous welfare standards
Versatility/scalability: Large body size allows for multiple wounds per animal and testing of full‐size devices/dressings	Species differences: Pigs have mostly apocrine sweat glands and fewer melanocytes
Advanced analytics compatibility: Pigs accommodate serial biopsies, high resolution imaging and multi‐omics approaches in vivo	Chronicity modelling challenges: Healthy pigs tend to heal wounds that would remain chronic in humans

### 5.2 Limitations

Despite their substantial translational value, porcine wound models have important limitations that should be considered when designing experiments and interpreting results. Foremost among these is the resource‐intensive nature of large‐animal research. Porcine studies require specialised housing, trained personnel, anaesthesia and ongoing veterinary support, all of which increase cost and logistical complexity. Animal size also introduces practical challenges related to handling, procedural consistency and prolonged monitoring, particularly in studies requiring repeated interventions or long‐term follow‐up.

In addition to practical considerations, biological differences between porcine and human skin remain important. Although porcine skin closely resembles human skin in many structural and functional aspects, differences in adnexal composition, including the relative absence of widespread eccrine sweat glands, may influence re‐epithelialisation dynamics and barrier restoration [133]. Variations in vascular architecture, thermoregulation and local immune responses may also affect specific aspects of wound healing. These distinctions are particularly relevant when interpreting mechanisms of epithelial regeneration and inflammatory signalling and underscore the need for careful endpoint selection.

A central limitation of porcine wound models is their intrinsic capacity for robust healing, even under conditions of experimental perturbation. Most wounds in pigs, including those subjected to ischaemia, infection or metabolic stress, ultimately close within weeks. As a result, porcine systems generally model delayed or impaired healing rather than true chronicity, in contrast to human chronic wounds, which may persist for months or years. Even advanced approaches (such as combined comorbidity models or chemically induced chronic‐like systems) reproduce selected aspects of impaired repair but do not fully replicate the long‐standing, multifactorial disease processes characteristic of clinical chronic wounds. This limitation is particularly relevant for chronic‐like models, which are intentionally engineered to delay healing but do not replicate the prolonged disease evolution seen in patients.

Experimental variability represents an additional limitation. Differences in pig breed, age, body size, skin characteristics and anatomical wound location can introduce heterogeneity in healing outcomes. Technical factors, including wound creation depth, device calibration, surgical technique and post‐operative care, further contribute to variability across studies. Maintaining dressings on active animals and ensuring consistency in treatment delivery can also be challenging. These factors highlight the importance of standardised protocols, operator training and rigorous reporting to enhance reproducibility and comparability between studies.

Finally, porcine models do not inherently capture the complex comorbidity landscape that underlies most human chronic wounds. Experimental animals are typically young and otherwise healthy, lacking conditions such as advanced vascular disease, long‐standing diabetes with associated neuropathy, ageing‐related immune dysfunction and cumulative environmental or mechanical stress. While individual comorbidities can be introduced experimentally, no single porcine model fully reproduces the integrated, long‐duration pathology observed in patients. Consequently, porcine wound models are best interpreted as highly informative but partial representations of human disease.

In summary, porcine wound models offer strong translational advantages but require careful consideration of their limitations. Recognition of practical constraints, species‐specific biology and challenges in modelling chronic disease is essential for appropriate study design and interpretation. When used alongside complementary experimental systems and clinical data, these models remain a powerful component of the translational wound‐healing research pipeline.

## 6 Future Directions and Innovations

Research efforts are increasingly focused on improving porcine wound models to address recognised limitations in modelling impaired and chronic‐like healing (Figure 3). A major area of development is the integration of combined injury paradigms that incorporate factors such as ischaemia, infection, metabolic dysfunction or chemically induced tissue perturbation. These approaches aim to better reflect the multifactorial nature of human chronic wounds, which rarely arise from a single pathological process. Although technically complex and potentially more variable, such combined systems may improve biologic relevance by reproducing interacting mechanisms that contribute to delayed repair.

Further refinement of chronic‐like wound induction strategies is also underway. These include chemically mediated approaches such as GA‐based systems, as well as models that combine systemic and local impairments to generate more persistent healing delay. Such platforms offer the advantage of creating reproducible, experimentally controlled chronic‐like states; however, they remain engineered systems and require broader validation across laboratories before they can be considered established standards.

Advances in molecular profiling and spatially resolved analyses are also transforming porcine wound research. Transcriptomic, proteomic, lipidomic and high‐resolution imaging approaches are increasingly being applied to define cellular and molecular changes across healing phases and wound regions [4, 33, 126]. These methods support linking conventional histologic or clinical outcomes with underlying biologic programs and may help identify pathways associated with impaired repair or treatment response. However, the interpretation of such high‐dimensional datasets depends on careful study design, rigorous annotation and reproducible analytical workflows.

Emerging applications of computational and machine learning approaches represent another promising direction. Recent porcine wound datasets that combine serial wound photography with transcriptomic profiling have been described as a resource for developing intelligent wound diagnostics and treatment algorithms [113]. In parallel, semi‐automated histological analysis has been applied in standardised porcine wound models to generate quantitative morphometric readouts of wound compartments, illustrating a practical route toward computationally enhanced outcome assessment. More broadly, image‐based analysis of wound progression may help identify predictive biomarkers or classify healing trajectories when paired with well‐annotated molecular datasets. However, these applications remain in relatively early stages in porcine systems and will require broader validation, standardised input data and carefully defined endpoints before routine translational use.

Recent work has also explored genetically engineered and immunologically modified porcine systems. Advances in CRISPR/Cas9 gene editing have enabled the development of pigs with targeted alterations in pathways relevant to metabolism, immune function and tissue repair. In parallel, immunodeficient or ‘humanised’ pigs engrafted with human cells have been proposed as platforms for studying human‐specific immune responses in a large‐animal setting [110, 111, 112]. While these systems remain at an early stage in wound‐healing research, they may ultimately provide valuable tools for modelling disease mechanisms and evaluating targeted therapies that cannot be adequately studied in conventional porcine models.

Finally, increasing attention is being directed toward understanding biologic variability within porcine systems, including differences between breeds, anatomical sites and local tissue environments, all of which may influence healing outcomes and scar formation. Further characterisation of these variables may improve model selection and experimental interpretation. At the same time, improved standardisation of wound creation methods, outcome measures and reporting practices remains essential for reproducibility and cross‐study comparison [31]. In summary, ongoing advances in model design, analytical technologies and biologic characterisation continue to expand the utility of porcine wound systems. However, many of these innovations remain in early stages and require further validation [111]. Careful integration of emerging approaches with well‐established models, standardised methodologies and clinically relevant endpoints will be essential to strengthen the translational value of porcine wound‐healing research.

In summary, ongoing advances in modelling approaches, analytical methods and translational strategies continue to expand the utility of porcine wound models. While challenges remain, particularly in modelling chronic disease states and integrating complex comorbidities, these developments are likely to enhance the relevance of porcine systems for preclinical research. Continued refinement and validation of these approaches will be essential to further strengthen their role in bridging experimental findings and clinical application.

## Conflicts of Interest

The authors declare no conflicts of interest.

## Data Availability

Data sharing not applicable to this article as no datasets were generated or analysed during the current study.
